# Risk factors for peptic ulcer bleeding one year after the initial episode

**DOI:** 10.1186/s13099-024-00669-x

**Published:** 2024-11-28

**Authors:** Yu-Xuan Peng, Wen-Pei Chang

**Affiliations:** 1 Department of Nursing, Taipei Veterans General Hospital, Taipei, Taiwan; 2 Department of Nursing, Shuang Ho Hospital, Taipei Medical University, No. 291, Zhongzheng Rd, Zhonghe, New Taipei City 23561 Taiwan; 3 School of Nursing, College of Nursing, Taipei Medical University, Taipei, Taiwan

**Keywords:** Peptic ulcer bleeding, Inpatient, Recurrent bleeding, Risk factor

## Abstract

**Background:**

Peptic ulcers are a common gastrointestinal disease that could cause death when combined with bleeding. The aim of this study was to identify risk factors for peptic ulcer bleeding (PUB) recurrence after the initial episode.

**Methods:**

This retrospective study analyzed medical records of PUB patients who were admitted through the emergency department between January 1, 2020, and December 31, 2022. A multivariate logistic regression model was used to identify independent risk factors predicting readmission due to recurrent PUB within one year.

**Results:**

A total of 775 PUB inpatient samples were collected, among which 172 and 603 were placed respectively in the readmission group and non-readmission group. Multivariate analysis indicated that PUB inpatients who were aged 70 or above (OR = 1.62, 95% CI: 1.06–2.47), had more severe ulcers (Forrest 1a, 1b, 2a, or 2b) (OR = 2.41, 95% CI:1.57–3.71), had a CCI score of 3 or higher (OR = 2.25, 95% CI:1.45–3.50), had a medical history of peptic ulcers (OR = 3.87, 95% CI:2.56–5.85), had a medical history of cardiovascular disease (CVD) (OR = 2.31, 95% CI:1.53–3.50), or had an international normalized ratio (INR) > 1.2 on admission (OR = 2.14, 95% CI:1.28–3.57) were respectively more likely to be readmitted within a year due to PUB than those who were under the age of 70, had less severe ulcers (Forrest 2c or 3), had a CCI score of less than 3, had no medical history of peptic ulcers, had no medical history of CVD, or had admission INR ≤ 1.2.

**Conclusion:**

This study confirmed that age (≥70 years), Forest classification (Forrest 1a, 1b, 2a, or 2b), multiple comorbidities, a medical history of peptic ulcers, a medical history of CVD, and admission INR > 1.2 were independent risk factors for patient readmission within a year due to recurrent PUB.

## Background

Upper gastrointestinal bleeding is a common clinical disease estimated to occur in approximately 80 to 150 people out of 100,000 people every year, with a mortality rate between 2% and 15% [1]. Approximately half of upper gastrointestinal bleeding occurrences are associated with peptic ulcers, which are a common chronic disease of the digestive tract; 80% of peptic ulcer bleeding (PUB) cases cease spontaneously while the remaining cases experience recurrent bleeding [2]. A common consequence of peptic ulcers that reach blood vessels is internal bleeding [3]. Ulcers can also penetrate the walls of the stomach or duodenum, causing severe abdominal pain and leading to life-threatening peritonitis [4]. When ulcers heal, the formation of scar tissue could partially or completely obstruct the pylorus, affecting the normal functions of the gastrointestinal tract and causing indigestion (Negash et al., 2022) [5]. Furthermore, untreated peptic ulcers, particularly those caused by *Helicobacter pylori* infections, increase the risk for gastric cancer [6], and chronic blood loss caused by peptic ulcers could induce iron-deficiency anemia, which leads to frequent discomfort in the form of fatigue and dizziness [7]. The consequences of the aforementioned conditions all severely affect patient quality of life and potentially result in severe complications or even death. Moreover, significant increases in medical expenses are concurrently incurred [8]. The timely diagnosis and treatment of peptic ulcers are therefore of utmost importance.

Peptic ulcers can be divided based on location into gastric ulcers and duodenal ulcers, both of which are susceptible to rupturing and bleeding, resulting in PUB. In rare cases, gastric ulcers are associated with malignant tumors and should thus be assessed with caution [9]. Note that shallower mucosal damage that has yet to penetrate the deeper tissues of the digestive tract is known as erosion [10]. As the risk for recurrent bleeding exists following PUB due to a multitude of factors [11], an in-depth investigation of risk factors is needed to reduce the incidence of recurrent bleeding.

Past studies have identified multiple aspects that influence the risk for recurrent bleeding in PUB patients within three days of their first endoscopy. Following a diagnosis of ulcers during endoscopy, the Forrest classification is used to classify the ulcers based on severity to determine the best course of treatment because larger or deeper ulcers are more prone to recurrent bleeding. Abnormalities in laboratory blood test results such as platelet (PLT) count, hemoglobin (Hgb) level, and white blood cell (WBC) count could reflect the physiological status and potential risk for bleeding of patients and are thus correlated with a risk for recurrent bleeding. Clinical symptoms such as shock, hematemesis, or melena are also associated with recurrent ulcer bleeding within the first three days [12]. In addition, past literature reports have indicated a number of risk factors correlated with hospitalization due to recurrent ulcer bleeding within the first 30 days, including age, previous gastrointestinal bleeding, clinical status, nutritional status, renal dysfunction, current use of antithrombotic agents and non-steroidal anti-inflammatory drugs (NSAIDs), and endoscopic findings [13–16].

Research has shown that the risk for recurrent bleeding in PUB patients after successful endoscopic treatment is highest in the first three days, ranging from about 30–55% [15, 17], and that the risk for recurrent bleeding within the first 30 days is approximately 18–49% [14, 18]. While early research reported a recurrent bleeding rate of 16–27% in PUB patients within 12 months [19, 20], , there have been no recent studies on recurrent bleeding rates that follow patients for this same time period. We therefore adopted a cohort study approach to not only elucidate whether PUB patients experience recurrent bleeding within a year but also evaluate the various factors associated with recurrent bleeding. Findings from this study are aimed at providing medical teams with a deeper understanding and assisting them in formulating more effective treatment plans and preventive measures, leading to better overall patient care by which to reduce the risk for recurrent bleeding in PUB patients.

## Methods

### Study population

We adopted a retrospective method and examined the hospitalization records of patients who were hospitalized via the emergency room due to PUB from a large teaching hospital in northern Taiwan in the three-year period from January 1, 2020, to December 31, 2022. We focused on adult patients over the age of 20 and followed their medical records for up to a year after discharge. Patients who were readmitted to the hospital within a year of the initial discharge due to bleeding ulcers were placed in the readmission group. Patients who were under the age of 20 or died during hospitalization were excluded. For patients who were hospitalized multiple times due to bleeding ulcers during our study period, only data from the first admission were counted; data from subsequent readmissions were not included in this study. Moreover, prior to data analysis, any patient with missing data in any of the variables had their data removed; that is, we employed complete case analysis, and only patients with no missing values were included in our analysis.

### Data collection

Definitions of the variables in this study are as follows:

Age: Conventionally, the elderly are defined as individuals over the age of 65 [21]. Researchers have further divided them into youngest-old, middle-old, and oldest-old, who are individuals respectively between 60 and 69 years old, between 70 and 79 years old, and 80 years old or above [22]. This study investigated a total of 775 patients. Grouped by age, there were 162 patients (20.9%) under the age of 60, 200 patients (25.8%) aged 60 to 69, 203 patients (26.2%) aged 70 to 79, and 210 patients (27.1%) aged 80 or over. In other words, over half of them were over the age of 60, showing that most of the study population were elderly individuals. Considering the fact that only 222 patients (28.6%) were under the age of 65, we chose 70 years old as the cutoff point in our analysis.
Body mass index (BMI): The World Health Organization recommends using BMI, which is calculated by dividing an individual’s weight in kilograms by the square of their height in meters, to gauge body weight [23]. We utilized BMI data from the date of admission.
Other background data: This included the following: patient gender, which was male or female; educational background, which was obtained from nursing assessment records on admission and divided into (1) senior high school or below and (2) college and university or above; employment status, which included employed, retired, or unemployed; religious beliefs, which included religious or not religious; and marital status, which included single, married, divorced, or widowed.
Daily habits: In the nursing assessment on admission, patients were asked whether they had a smoking habit. Following an affirmative response, they were also asked how many years they had been smoking and the number of cigarettes that they smoked per day. They were also asked whether they had smoked in the past and, if so, for how long they had quit. In addition, the patients were asked on admission whether they had an alcohol drinking habit. Following an affirmative response, they were asked how often they drank, which was daily, weekly, or occasionally, to assess the regularity of their drinking. The nurses also recorded how much alcohol they drank each time to quantify their drinking behavior (e.g., three times a week and two glasses each time).
Symptoms on admission: These refer to the primary complaints commonly observed in PUB patients upon admission to the emergency department, including pain score, hematochezia, melena, mixed hematochezia and melena, hematemesis or coffee ground vomiting, and abdominal pain as well as shock, weakness, dizziness, or dyspnea. The pain score was collected from their medical records, which used the numeric rating scale from 0 to 10 to quantify the severity of pain, with 0 indicating no pain and 10 indicating the most severe pain. The numeric rating scale is highly sensitive with an intraclass correlation coefficient of 0.95 [24].
Endoscopy results: These refer to findings indicated in endoscopy reports from before the current hospitalization and treatment. The time at which endoscopies were taken was dependent on the patient’s clinical conditions. Upon a substantial drop in the patient’s hemoglobin levels, a clinical physician immediately arranged for an endoscopy. We therefore did not collect data regarding the time period between admission and endoscopy as it was directly associated with the patient’s clinical conditions. The severity of ulcers was determined using the Forrest classification in the report [25], which included the following from severe to mild: 1a (spurting hemorrhage), 1b (oozing hemorrhage), 2a (visible vessel), 2b (adherent clot), 2c (flat pigmented haematin on ulcer base), and 3 (lesion that has ceased bleeding). Patients are usually treated according to the guidelines and protocols of the treating hospital [26]. In general, patients with Forrest 1a and 1b peptic ulcers are in an acute active bleeding state and require immediate endoscopic treatment. Bleeding is generally stopped using hemostatic agents, thermal coagulation, or vascular clipping. Once bleeding ceases, proton pump inhibitors (PPIs) are administered for adjuvant therapy to stabilize the ulcers, promote healing, and prevent recurrent bleeding [27]. Patients with Forrest 2a and 2b peptic ulcers do not have active bleeding but are at high risk for bleeding. Thrombus removal or hemostatic measures are usually performed under endoscopy followed by thermal treatment or clipping to prevent recurrent bleeding, after which PPIs are prescribed [27]. In patients with Forrest 2c and 3 peptic ulcers, bleeding has ceased, and they are not at risk for bleeding. Interventional therapy under an endoscope is not required, and the only treatment necessary is PPIs to inhibit gastric acid secretion and promote ulcer healing [28]. In Taiwan, the benefit provisions of the National Health Insurance regarding PPIs for patients with bleeding peptic ulcers are as follows. After therapeutic endoscopic treatment for acute bleeding ulcers, an intravenous bolus of PPIs is administered during the acute bleeding phase, followed by a continuous intravenous infusion at a rate of 8 mg/hour for 72 h. PPI therapy can be switched from intravenous to oral as soon as the bleeding stops [29, 30]. Note that according to the benefit provisions of Taiwan’s National Health Insurance, physicians can prescribe PPI treatments for at least four months for high-risk patients (e.g., age over 65, medical history of peptic ulcers, long-term use of NSAIDs or aspirin, use of anticoagulants, chronic kidney disease (CKD), or liver disease) and patients with certain complications (e.g., peptic ulcer perforation, pyloric stenosis, and scarring) [31]. In the present study, if a patient had ulcers in multiple locations, we focused on the most severe ones. Consistent with other studies, we categorized Forrest 1a, 1b, 2a, and 2b as high-risk lesions and Forrest 2c and 3 as low-risk lesions [25, 32].
Charlson Comorbidity Index (CCI): This is a weighted total score of four specific categories of diseases. One point is given for myocardial infarction, congestive heart failure, peripheral vascular disease, cerebrovascular disease, dementia, chronic pulmonary disease, connective tissue disease, ulcerative disease, mild liver disease, and diabetes. Two points are given for hemiplegia, moderate or severe renal disease, end-stage diabetes with organ damage, tumors, leukemia, and lymphoma. Three points are given for moderate or severe liver cancer. Six points are given for acquired immune deficiency syndrome [33]. A higher total CCI indicates a more severe comorbid condition and poorer health. A CCI from 0 to 2 means that the patient does not have chronic diseases or only has mild comorbidities. A CCI of 3 or higher indicates moderate or more severe comorbidities [34]. In this study, we divided the patients into two groups: those with a CCI score of 0–2 and those with a CCI score ≥3.
Medical history of relevant diseases: Hospitalization records were reviewed to identify patients with peptic ulcers, cardiovascular disease (CVD), diabetes, or CKD.
  (9) Use of antithrombotic agents: Collectively called antithrombotic agents, antiplatelet and anticoagulant drugs primarily prevent thrombosis by inhibiting platelet aggregation (e.g., antiplatelet agents) or slowing down key enzymes in the coagulation process (e.g., anticoagulants). This is crucial in the prevention and treatment of many types of CVDs [35]. We searched the medical records of the patients for records of any anticoagulants (e.g., pradaxa, orfarin, xarelto, eliquis, and lixiana) and antiplatelet agents (e.g., clopidogrel, thrombifree, bokey, aspirin, dipyridamole, throne, licodin, pletaal, brilinta, and forflow) that they may have regularly taken within the past three months.
Use of NSAID analgesics: Although NSAIDs are not typical antiplatelet drugs, they possess antiplatelet effects [36]. We therefore searched the medical records of the patients for records of any NSAID analgesics (e.g., mobic, voren, cataflam, potarlon, vortagen, eunac, votan, celecoxib, and diclofenac) that they may have regularly taken within the past three months.
(11) Relevant pre-admission blood test results: These included white blood cell (WBC) count, prothrombin time (PT), international normalized ratio (INR), hemoglobin (Hgb) level, hematocrit (Hct), platelet (PLT) count, and creatinine (Cr). In accordance with the literature, we divided the patients into two groups for each parameter: >10,000/µl and ≤10,000/µl for WBC, > 14.5 s and ≤14.5 s for PT, > 1.2 and ≤1.2 for INR, ≥10 g/dL and < 10 g/dL for Hgb, ≥31.5% and < 31.5% for Hct, ≥150,000/µl and < 150,000/µl for PLT, and > 1.3 mg/dL and ≤1.3 mg/dL for Cr.

### Research privacy and confidentiality of data

We obtained approval from the institutional review board of the study hospital. Based on ethical considerations, medical record numbers were used to string together different data fields in the collected data. Upon completion, the medical record numbers were replaced with serial numbers in order to protect the privacy of research subjects.

### Statistics

The number of required samples was estimated using G-power 3.1.9.7 for logistic regression analysis. Our settings included a type-I error of 5% and power of 95%. Due to the limited amount of related research, an odds ratio (OR) of 1.8 was chosen as our hypothetical value. This was based on existing literature suggesting that the risk for gastrointestinal bleeding in patients with CKD was 1.8 times higher than a control group with normal kidney function [37]. We therefore set the OR at 1.8, with at least 719 samples needed.

Following data collection, we used SPSS 25.0 for Windows to conduct our statistical analyses. The descriptive analysis included frequency distribution, percentage, mean, and standard deviation. We then compared the data of the variables using t-tests or chi-square tests. Next, we performed binary logistic regression analysis with the basic attributes of the patients (including age, gender, educational background, employment status, religion, marital status, and BMI), daily habits (including smoking and drinking habits), symptoms upon admission (including pain score, hematochezia, melena, mixed hematochezia and melena, hematemesis or coffee ground vomiting, and abdominal pain as well as shock, weakness, dizziness, or dyspnea), endoscopy results (including location and severity of ulcers), other comorbidities or drug use (including CCI, medical history involving peptic ulcers, CVD, diabetes, CKD, use of antithrombotic agents, and use of NSAID analgesics), and pre-admission blood test results (including WBC, PT, INR, Hgb, Hct, PLT, and Cr) as the independent variables to conduct predictive power analysis regarding whether the patients would be re-hospitalized due to PUB. In the selection of predictors, we first adopted bivariate analysis to identify variables with significant correlation and then performed predictive power analysis regarding whether the patients would be re-hospitalized due to PUB using the correlated variables and the multivariate model.

## Results

### PUB inpatient variable data

We originally collected data from 793 patients, among which 18 were removed due to missing values. We compared the characteristics of these 18 patients with the remaining 775 patients and found no significant differences in the distribution in age, gender, or Forrest classification. Therefore, removing the samples with missing data did not significantly affect the representativeness of our patient population. For our study, 775 patients were analyzed (172 in the readmission group and 603 in the non-readmission group). Their mean age was 69.84 years old (*SD* = 13.97) with a mean BMI of 24.86 kg/m^2^ (*SD* = 4.76). Over half of the inpatients were male (63.7%), and the main symptom upon admission was melena (65.7%). The most common endoscopy results were 1a, 1b, 2a, or 2b ulcers (64.6%). Among the inpatients, 38.3% had CCI ≥3, 24.6% had a medical history of peptic ulcers, 41.2% had a medical history of CVD, 53.8% had PT > 14.5 s, 27.0% had INR > 1.2, 57.5% had Hgb levels < 10 g/dL, 66.5% had Hct < 31.5%, and 38.1% had Cr > 1.3 mg/dL (Table 1).

**Table 1:** Analysis of differences between basic data distributions of PUB inpatients who were and were not readmitted within a year due to PUB (including frequency and percentage of original samples with missing data for each variable)

Variable	All	Readmission	Non-readmission	*p*	Missing Data (*n*, %)
	*N =* 775	*n* = 172	*n* = 603		
Basic attributes					
Age, mean (*SD*)	69.84 (13.97)	74.61 (14.70)	68.48 (14.27)	< 0.001	0 (0%)
BMI, mean (*SD*)	24.86 ( 4.76)	24.81 ( 5.35)	24.87 ( 4.59)	0.885	6 (0.8%)
Gender: male, *n* (%)	494 (63.7)	112 (65.1)	382 (63.3)	0.671	0 (0%)
Educational background: senior high school or below	666 (85.9)	152 (88.4)	514 (85.2)	0.297	0 (0%)
Employment status: employed, *n* (%)	245 (31.6)	44 (25.6)	201 (33.3)	0.054	0 (0%)
Religion: religious, *n* (%)	461 (59.5)	106 (61.6)	355 (58.9)	0.516	0 (0%)
Marital status: married, *n* (%)	541 (69.8)	115 (66.9)	426 (70.6)	0.340	0 (0%)
Daily habits, *n* (%)					
Daily smoking	181 (23.4)	47 (27.3)	134 (22.2)	0.163	0 (0%)
Daily drinking	57 ( 7.4)	13 ( 7.6)	44 ( 7.3)	0.908	0 (0%)
Symptoms upon admission					
Pain score, mean (*SD*)	0.74 (1.82)	0.65 (1.72)	0.77 (1.84)	0.417	0 (0%)
Hematochezia, *n* (%)	31 ( 4.0)	11 ( 6.4)	20 (3.3)	0.069	0 (0%)
Melena, *n* (%)	509 (65.7)	121 (70.3)	388 (64.3)	0.144	0 (0%)
Hematochezia and melena mix, *n* (%)	29 ( 3.7)	3 ( 1.7)	26 (4.3)	0.118	0 (0%)
Hematemesis or coffee ground vomiting, *n* (%)	222 (28.6)	44 (25.6)	178 (29.5)	0.314	0 (0%)
Abdominal pain, *n* (%)	123 (15.9)	24 (14.0)	99 (16.4)	0.435	0 (0%)
Shock, weakness, dizziness, or dyspnea, *n* (%)	161 (20.8)	36 (20.9)	125 (20.7)	0.954	0 (0%)
Endoscopy results, *n* (%)					
Ulcer location: both stomach and duodenum	168 (21.7)	28 (16.3)	140 (23.2)	0.051	0 (0%)
Ulcer severity: Forrest 1a, 1b, 2a, or 2b	501 (64.6)	130 (75.6)	371 (61.5)	0.001	0 (0%)
Other comorbidities or use of relevant drugs, *n* (%)					
CCI ≥ 3	297 (38.3)	107 (62.2)	190 (31.5)	<0.001	0 (0%)
Medical history of peptic ulcers: yes	191 (24.6)	79 (45.9)	112 (18.6)	< 0.001	0 (0%)
Medical history of CVD: yes	319 (41.2)	109 (63.4)	210 (34.8)	< 0.001	0 (0%)
Medical history of DM: yes	274 (35.4)	63 (36.6)	211 (35.0)	0.692	0 (0%)
Medical history of CKD: yes	105 (13.5)	40 (23.3)	65 (10.8)	< 0.001	0 (0%)
Use of antithrombotic agents (anticoagulants and antiplatelet agents)	317 (40.9)	70 (40.7)	247 (41.0)	0.950	0 (0%)
Use of NSAID analgesics	272 (35.1)	68 (39.5)	204 (33.8)	0.167	0 (0%)
Pre-admission blood test results, *n* (%)					
WBC > 10,000/µl	425 (54.8)	90 (52.3)	335 (55.6)	0.453	9 (1.1%)
PT > 14.5 s	417 (53.8)	106 (61.6)	311 (51.6)	0.020	5 (0.6%)
INR > 1.2	209 (27.0)	67 (39.0)	142 (23.5)	< 0.001	5 (0.6%)
Hgb < 10 g/dL	446 (57.5)	118 (68.6)	328 (54.4)	0.001	0 (0%)
Hct < 31.5%	515 (66.5)	133 (77.3)	382 (63.3)	0.001	0 (0%)
PLT < 150,000/µl	84 (10.8)	24 (14.0)	60 (10.0)	0.136	0 (0%)
Cr > 1.3 mg/dL	295 (38.1)	80 (46.5)	215 (35.7)	0.010	11 (1.4%)

*Abbreviations* BMI, body mass index; CCI, Charlson comorbidity index; CVD, cardiovascular disease; DM, diabetes mellitus; CKD, chronic kidney disease; PT, prothrombin time; INR, international normalized ratio; NSAID, non-steroidal anti-inflammatory drug; WBC, white blood cell; Hgb, hemoglobin; Hct, hematocrit; PLT, platelet; Cr, creatinine; *SD*, standard deviation

### Comparison of patients in readmission group and non-readmission group

A total of 172 inpatients were readmitted within a year, whereas 603 inpatients were not readmitted. A comparison of these two groups of inpatients revealed significant differences in mean age (*p* < .001), ulcer severity in their endoscopy results (*p* = .001), CCI ≥3 (*p* < .001), medical history of peptic ulcers (*p* < .001), medical history of CVD (*p* < .001), medical history of CKD (*p* < .001), PT > 14.5 s (*p* = .020), INR > 1.2 (*p* < .001), Hgb < 10 g/dL (*p* = .001), Hct < 31.5% (*p* = .001), and Cr > 1.3 mg/dL (*p* = .010) (Table 1).

### Predictors of readmission within a year due to PUB

Table 2 presents the binary logistic regression analysis performed with age, ulcer severity, CCI ≥3, medical history of peptic ulcers, medical history of CVD, medical history of CKD, PT > 14.5 s, INR > 1.2, Hgb < 10 g/dL, Hct < 31.5%, and Cr > 1.3 mg/dL as independent variables. The results showed that the regression coefficients of age 70 or above (OR = 2.94, *p* < .001), more severe ulcers (1a, 1b, 2a, or 2b) (OR = 1.94, *p* = .001), CCI ≥3 (OR = 3.58, *p* < .001), medical history of peptic ulcers (OR = 3.72, *p* < .001), medical history of CVD (OR = 3.24, *p* < .001), medical history of CKD (OR = 2.51, *p* < .001), PT > 14.5 s (OR = 1.51, *p* = .020), INR > 1.2 (OR = 2.07, *p* < .001), Hgb < 10 g/dL (OR = 1.83, *p* = .001), Hct < 31.5% (OR = 1.97, *p* = .001), and Cr > 1.3 mg/dL (OR = 1.57, *p* = .010) were significant, thereby indicating that these variables were associated with readmission within a year due to PUB. We therefore conducted multivariate logistic regression analysis using these variables. The results of the multivariate logistic regression analysis indicated that only age 70 or above (OR = 1.62, *p* = .025), high-risk ulcers (1a, 1b, 2a, or 2b) (OR = 2.41, *p* < .001), CCI ≥3 (OR = 2.25, *p* < .001), medical history of peptic ulcers (OR = 3.87, *p* < .001), medical history of CVD (OR = 2.31, *p* < .001), and INR > 1.2 (OR = 2.14, *p* = .004) could be used to predict the risk for readmission within a year due to PUB.

**Table 2:** Logistic regression predicting readmission within a year due to PUB

Variable	bivariate		multivariate	
	OR (95% CI)	*p*	OR (95% CI)	*p*
Age 70 or above^a^	2.49 (1.73–3.59)	< 0.001	1.62 (1.06–2.47)	0.025
Endoscopy results: ulcer severity Forrest 1a, 1b, 2a, or 2b^b^	1.94 (1.32–2.84)	0.001	2.41 (1.57–3.71)	< 0.001
CCI ≥ 3	3.58 (2.51–5.09)	< 0.001	2.25 (1.45–3.50)	< 0.001
Medical history of peptic ulcers: yes^c^	3.72 (2.59–5.36)	< 0.001	3.87 (2.56–5.85)	< 0.001
Medical history of CVD: yes^c^	3.24 (2.28–4.61)	< 0.001	2.31 (1.53–3.50)	< 0.001
Medical history of CKD: yes^c^	2.51 (1.62–3.89)	< 0.001	1.41 (0.79–2.54)	0.248
Pre-admission blood test result: PT > 14.5 s	1.51 (1.07–2.13)	0.020	0.76 (0.46–1.26)	0.288
Pre-admission blood test result: INR > 1.2	2.07 (1.45–2.97)	< 0.001	2.14 (1.28–3.57)	0.004
Pre-admission blood test result: Hgb < 10 g/dL	1.83 (1.28–2.63)	0.001	1.43 (0.72–2.83)	0.304
Pre-admission blood test result: Hct < 31.5%	1.97 (1.33–2.92)	0.001	1.19 (0.56–2.50)	0.652
Pre-admission blood test result: Cr > 1.3 mg/dL	1.57 (1.11–2.21)	0.010	0.85 (0.54–1.34)	0.483

^a^(Reference group: age under 70)

^b^(Reference group: 2c or 3)

^c^(Reference group: History of related medical disease: no)

*Abbreviations* CCI, Charlson comorbidity index; CVD, cardiovascular disease; CKD, chronic kidney disease; PT, prothrombin time; INR, international normalized ratio; Hgb, hemoglobin; Hct, hematocrit; Cr, creatinine; OR, odds ratio; CI, confidence interval

## Discussions

Our findings reveal that after controlling factors that could influence the readmission of PUB inpatients within a year, those who were aged 70 or above as well as those who had more severe ulcers (Forrest 1a, 1b, 2a, or 2b), multiple comorbidities (CCI score ≥3), a medical history of peptic ulcers, a medical history of CVD, or INR > 1.2 were respectively 1.62, 2.41, 2.25, 3.87, 2.31, and 2.14 times more likely to be readmitted within a year due to PUB than those who were under the age of 70 as well as those who had less severe ulcers (Forrest 2c or 3), fewer comorbidities, no medical history of peptic ulcers, no medical history of CVD, or INR ≤1.21.

This study employed a retrospective research design where the data of the patient population were derived from actual clinical settings from the hospital to which the patients had been admitted. Although this could have introduced some selection bias, which is characteristic of retrospective research, the patient population was still representative of the population as a whole to a sufficient extent [38]. The clinical backgrounds of the patients in this study, such as older age and increased complications, were inherently associated with a higher risk for recurrent ulcer bleeding and readmission. In other words, patients who are older or have more complications are deemed high-risk groups for recurrent ulcer bleeding and readmission [39]. Moreover, a large proportion of the patient group in this study were taking antithrombotic agents, which reflects that suffering from CVDs or other comorbidities increases the risk for recurrent ulcer bleeding and readmission [40]. Thus, these findings have significant clinical implications for patients who are older in age or suffering from CVDs or other complications.

In the face of physiological aging, the gastrointestinal functions of elderly patients gradually weaken, rendering the mucosa more susceptible to damage and increasing the risk for ulcer formation and bleeding [41]. As tissue repair and regeneration capabilities decline with age, repairing damage from ulcers is even more difficult and thereby increases the risk for recurrent bleeding [42]. The results of this study found that age 70 or above is a crucial risk factor for readmission to hospital within a year due to PUB, which indicates that elderly individuals over this age are more prone to recurrent ulcer bleeding, due perhaps to these physiological changes.

The risk for recurrent PUB is also increased by the presence of chronic diseases. For instance, diabetes could impair the nervous system and blood vessels, thereby affecting normal gastrointestinal functions. Research in rodent models has demonstrated that diabetes increases susceptibility to gastric injury and inhibits the healing of damaged gastric mucosa [43]. From Taiwan’s National Health Insurance Research Database, Liang et al. divided 1,229 patients who were admitted for treatment following endoscopic hemostasis for PUB into a CKD group (*n* = 184) and a non-CKD group (*n* = 1045) [44]. After a ten-year follow-up period, they discovered that the recurrent bleeding rate after discharge in the CKD group was higher than that in the non-CKD group (11.96% vs. 6.32%, *p* = .006). Blood supply to the gastrointestinal tract is also affected by coronary heart disease and heart failure due to poor blood circulation, which increases the risk for recurrent PUB [45]. Elevations in blood vessel pressure from declines in renal function lead to the accumulation of waste and fluid in the body and render mucosal blood vessels in the gastrointestinal tract more susceptible to damage, further increasing the risk for ulcer formation and bleeding [46]. In particular, patients with end-stage renal disease requiring hemodialysis are often at higher risk due to platelet dysfunction, which negatively influences the adhesiveness of platelets to the vascular walls of bleeding ulcers [47]. Diseases such as cirrhosis or hepatitis affect liver function and impact both coagulation as well as blood supply to the gastrointestinal tract [48]. Cirrhosis is associated with portal hypertension and increases the risk for esophageal and gastric varices, all of which are risk factors for PUB [49]. In a prospective observational study conducted at two hospitals in Norway, Romstad et al. demonstrated that out of the comorbidities CVD, renal failure, diabetes, and cirrhosis, CVD was the most critical risk factor for PUB [32]. Research has also indicated that negative synergism occurs when two or more of the above chronic diseases are present in a patient. Infante et al. investigated 103 patients with PUB using CCI to determine whether the presence of comorbidities was associated with recurrent bleeding [50]. Their results revealed that patients with CCI of 2 or 3 were at greater risk for recurrent bleeding within 30 days than were patients with CCI of 1 (RR = 1.5; 95% CI: 0.25–8.97, *p* = .006). After controlling other risk factors, we demonstrated that CCI ≥3 is indeed a predictor of the readmission of PUB inpatients within a year due to PUB.

This study found that CVD is also a risk factor for the readmission of patients within a year due to recurrent PUB, due perhaps to the use of antithrombotic agents by CVD patients increasing the risk for bleeding in the digestive tract mucosa [51]. CVD patients are often hemodynamically unstable, which affects the blood supply to their gastrointestinal tract and makes the gastrointestinal mucosa more susceptible to damage [52]. Moreover, CVD is associated with chronic low-grade inflammation, in which systemic inflammatory responses increase the susceptibility of the gastrointestinal mucosa and perturb ulcer repair processes [53]. These CVD-related factors collectively render peptic ulcers more likely to recur after bleeding, thereby significantly increasing the risk for recurrent bleeding. In addition, patients with INR > 1.2 after their initial episode of gastrointestinal bleeding may have difficulty forming effective blood clots to prevent bleeding, such that this poor coagulation further exacerbates the likelihood of ulcers re-bleeding [54]. Thus, abnormal INR on admission is an indicator of an increased risk for recurrent bleeding in peptic ulcers and should be given special attention in clinical practice.

In terms of severe peptic ulcers, damage to nearby blood vessels increases the vulnerability of patients to more ulcers and thus the risk for further bleeding [55]. Romstad et al. analyzed 543 PUB patients and found that those with more severe ulcers (Forrest 1a, 1b, 2a, or 2b) are more likely to experience recurrent bleeding than those with less severe ulcers (Forrest 2c or 3) (17.2% vs. 4.6%, *p* < .001) [32]. This may be due to the fact that more severe peptic ulcers generally indicate a wider range of tissue damage that reaches or penetrates the mucosa, making the healing process even more complicated and increasing the risk for developing additional ulcers [56]. The results of our study further reveal that ulcer severity is an important risk factor for readmission within a year due to PUB. We also found that patients who had previously suffered PUB were 3.49 times more likely to require hospitalization and treatment within a year due to recurrent bleeding than those with no medical history of PUB. One possible explanation is the presence of prior damage in the gastrointestinal tissue, which increases the risk for recurrent PUB [57].

### Limitations

The data collected from the medical records in this study were limited by the completeness and accuracy of past medical records. We could only analyze the data included in the records and did not have access to comprehensive patient information. Thus, this is a limitation of our study. Furthermore, past studies have indicated that a high correlation exists between post-discharge use of drugs such as NSAIDs and antithrombotic agents with readmission due to recurrent PUB [38, 58]. However, as our study was a retrospective study, we could not collect all of the frequencies and dosages of medication taken by the patients before hospitalization and after discharge. Despite the absence of data on post-discharge drug use, predictors of readmission due to recurrent PUB were still identified in this study using other variables, such as age, comorbidities, and CVD, all of which could be used to evaluate the risk for readmission due to recurrent PUB with a high degree of confidence.

## Conclusions

We conducted a retrospective cohort study and discovered that for PUB inpatients, age 70 or above, Forest classification, multiple comorbidities, a medical history of peptic ulcers, a medical history of CVD, and admission INR > 1.2 were risk factors of readmission within a year due to PUB. To prevent recurrent PUB, we suggest the following to medical teams. More attention should be given to the health conditions of elderly patients as more severe ulcers could indicate a higher risk for recurrent bleeding. Patients whose initial PUB is under control still require monitoring and treatment for longer periods of time to reduce the risk for recurrent bleeding. Finally, the physical conditions of PUB patients with multiple comorbidities are complex, particularly PUB patients who have a medical history of CVD or are found to have poor coagulation functions on admission. Therefore, medical teams should consider comprehensive treatment plans based on the overall health conditions of the patients to prevent recurrent PUB.

## Data Availability

The data that support the findings of this study are available on request from the corresponding author. The data are not publicly available due to privacy or ethical restrictions.

## References

[1] Antunes C, Copelin IIEL. Upper gastrointestinal bleeding. [Updated 2023 Apr 7]. StatPearls. 2023; 4: 7. Cited 31 Dec 2023. Available from URL: https://www.ncbi.nlm.nih.gov/books/NBK470300/

[2] Petrik P, Brašiškienė S, Petrik E. Characteristics and outcomes of gastroduodenal ulcer bleeding: a single-centre experience in Lithuania. Prz Gastroenterol. 2017;12(4):277–85. 29358997 10.5114/pg.2017.72103 PMC5771452

[3] Bitar SM, Moussa M. The risk factors for the recurrent upper gastrointestinal hemorrhage among acute peptic ulcer disease patients in Syria: a prospective cohort study. Ann Med Surg (Lond). 2022;74:103252. 35106151 10.1016/j.amsu.2022.103252 PMC8784635

[4] Weledji EP. An overview of gastroduodenal perforation. Front Surg. 2020;7:573901. 33240923 10.3389/fsurg.2020.573901 PMC7680839

[5] Negash S, Jembere T, Abera G, Kedir E, Eshetu B. Gastric outlet obstruction due to peptic ulcer disease in a 5 years-old female child. Case report. June 23, 2022. Int J Surg Case Rep. 2023;105:108086. 37018952 10.1016/j.ijscr.2023.108086 PMC10112143

[6] Paragomi P, Dabo B, Pelucchi C, et al. The association between peptic ulcer disease and gastric cancer: results from the stomach Cancer Pooling (StoP) Project Consortium. Cancers (Basel). 2022;14(19):4905. 36230828 10.3390/cancers14194905 PMC9563899

[7] Al Mutawa OA, Izhari MA, Alharbi RA, et al. Helicobacter pylori (H. Pylori) infection-associated anemia in the Asir Region, Saudi Arabia. Diagnostics (Basel). 2023;13(14):2404. 37510148 10.3390/diagnostics13142404 PMC10378611

[8] Lanas A, Dumonceau JM, Hunt RH, et al. Non-variceal upper gastrointestinal bleeding. Nat Rev Dis Primers. 2018;4:18020. 29671413 10.1038/nrdp.2018.20

[9] Barbu AM, Iordache S. Gastric and duodenal ulcers. In: Săftoiu A, editor. Pocket guide to advanced endoscopy in gastroenterology. Cham: Springer; 2023. 10.1007/978-3-031-42076-4_23.

[10] Deng Z, Zhu J, Ma Z, Yi Z, Tuo B, Li T, Liu X. The mechanisms of gastric mucosal injury: focus on initial chief cell loss as a key target. Cell Death Discov. 202;9(1):29. 10.1038/s41420-023-01318-z PMC9873797 36693845

[11] Nazarian S, Lo FPW, Qiu J, Patel N, Lo B, Ayaru L. Development and validation of machine learning models to predict the need for haemostatic therapy in acute upper gastrointestinal bleeding. Ther Adv Gastrointest Endosc. 2024;17:26317745241246899. 38712011 10.1177/26317745241246899 PMC11071626

[12] Lai Y, Xu Y, Zhu Z, et al. Development and validation of a model to predict rebleeding within three days after endoscopic hemostasis for high-risk peptic ulcer bleeding. BMC Gastroenterol. 2022;22(1):64. 35164682 10.1186/s12876-022-02145-9 PMC8843020

[13] Bitar SM, Moussa M. The risk factors for the recurrent upper gastrointestinal hemorrhage among acute peptic ulcer disease patients in Syria: a prospective cohort study. Ann Med Surg. 2022;74:103252. 10.1016/j.amsu.2022.103252 PMC8784635 35106151

[14] Camus M, Jensen DM, Kovacs TO, Jensen ME, Markovic D, Gornbein J. Independent risk factors of 30-day outcomes in 1264 patients with peptic ulcer bleeding in the USA: large ulcers do worse. Aliment Pharmacol Ther. 2016;43(10):1080–9. 27000531 10.1111/apt.13591 PMC4837138

[15] El Ouali S, Barkun A, Martel M, Maggio D. Timing of rebleeding in high-risk peptic ulcer bleeding after successful hemostasis: a systematic review. Can J Gastroenterol Hepatol. 2014;28(10):543–8. 25390616 10.1155/2014/324967 PMC4234354

[16] Ogiyama H, Tsutsui S, Murayama Y, et al. Renal dysfunction is an independent risk factor for rebleeding after endoscopic hemostasis in patients with peptic ulcer bleeding. Turk J Gastroenterol. 2021;32(8):622–30. 34528875 10.5152/tjg.2021.20202 PMC8975289

[17] Eisner F, Hermann D, Bajaeifer K, Glatzle J, Königsrainer A, Küper MA. Gastric ulcer complications after the introduction of proton pump inhibitors into clinical routine: 20-year experience. Visc Med. 2017;33(3):221–6. 28785572 10.1159/000475450 PMC5527226

[18] Bini EJ, Cohen J. Endoscopic treatment compared with medical therapy for the prevention of recurrent ulcer hemorrhage in patients with adherent clots. Gastrointest Endosc. 2003;58(5):707–14. 14595306 10.1016/s0016-5107(03)02014-5

[19] Jaspersen D, Koerner T, Schorr W, Brennenstuhl M, Raschka C, Hammar CH. Helicobacter pylori eradication reduces the rate of rebleeding in ulcer hemorrhage. Gastrointest Endosc. 1995;41:5–7. 7698624 10.1016/s0016-5107(95)70267-9

[20] Rokkas T, Karameris A, Mavrogeorgis A, Rallis E, Giannikos N. Eradication of Helicobacter pylori reduces the possibility of rebleeding in peptic ulcer disease. Gastrointest Endosc. 1995;41(1):1–4. 7698617 10.1016/s0016-5107(95)70266-0

[21] Singh S, Bajorek B. Defining ‘elderly’ in clinical practice guidelines for pharmacotherapy. Pharm Pract. 2014;12(4):489. 10.4321/s1886-36552014000400007 PMC4282767 25580172

[22] Lee SB, Oh JH, Park JH, Choi SP, Wee JH. Differences in youngest-old, middle-old, and oldest-old patients who visit the emergency department. Clin Exp Emerg Med. 2018;5(4):249–55. 30571903 10.15441/ceem.17.261 PMC6301865

[23] Fryar CD, Kruszon-Moran D, Gu Q, Ogden CL. Mean body weight, height, waist circumference, and body mass index among adults: United States, 1999–2000 through 2015–2016. Natl Health Stat Rep. 2018;(122):1–16. 30707668

[24] Alghadir AH, Anwer S, Iqbal A, Iqbal ZA. Test-retest reliability, validity, and minimum detectable change of visual analog, numerical rating, and verbal rating scales for measurement of osteoarthritic knee pain. J Pain Res. 2018;11:851–6. 29731662 10.2147/JPR.S158847 PMC5927184

[25] Yen HH, Wu PY, Wu TL, et al. Forrest classification for bleeding peptic ulcer: a new look at the old endoscopic classification. Diagnostics. 2022;12(5):1066. 35626222 10.3390/diagnostics12051066 PMC9139956

[26] Barkun AN, Almadi M, Kuipers EJ, et al. Management of nonvariceal upper gastrointestinal bleeding: guideline recommendations from the International Consensus Group. Ann Intern Med. 2019;171(11):805–22. 31634917 10.7326/M19-1795 PMC7233308

[27] ASGE technology committee, Parsi MA, Schulman AR, et al. Devices for endoscopic hemostasis of nonvariceal GI bleeding (with videos). VideoGIE. 2019;4(7):285–99. 31334417 10.1016/j.vgie.2019.02.004 PMC6616320

[28] Yang EH, Wu CT, Kuo HY, Chen WY, Sheu BS, Cheng HC. The recurrent bleeding risk of a Forrest IIc lesion at the second-look endoscopy can be indicated by high Rockall scores ≥ 6. Surg Endosc. 2020;34(4):1592–601. 31222633 10.1007/s00464-019-06919-3 PMC7223755

[29] Wu CY, Wu CH, Wu MS, et al. A nationwide population-based cohort study shows reduced hospitalization for peptic ulcer disease associated with H pylori eradication and proton pump inhibitor use. Clin Gastroenterol Hepatol. 2009;7(4):427–31. 19264578 10.1016/j.cgh.2008.12.029

[30] Chang SS, Hu HY. Helicobacter pylori eradication within 120 days is associated with decreased complicated recurrent peptic ulcers in peptic ulcer bleeding patients. Gut Liver. 2015;9(3):346–52. 25167793 10.5009/gnl13451 PMC4413968

[31] Lin XH, Lin CC, Wang YJ, et al. Risk factors of the peptic ulcer bleeding in aging uremia patients under regular hemodialysis. J Chin Med Assoc. 2018;81(12):1027–32. 29778548 10.1016/j.jcma.2018.03.007

[32] Romstad KK, Detlie TE, Søberg T, et al. Gastrointestinal bleeding due to peptic ulcers and erosions - a prospective observational study (BLUE study). Scand J Gastroenterol. 2020;55(10):1139–45. 32931710 10.1080/00365521.2020.1819405

[33] Charlson ME, Pompei P, Ales KL, MacKenzie CR. A new method of classifying prognostic comorbidity in longitudinal studies: development and validation. J Chronic Dis. 1987;40(5):373–83. 3558716 10.1016/0021-9681(87)90171-8

[34] Bhattacharjee HK, Kaviyarasan MP, Singh KJ, et al. Age adjusted Charlson comorbidity index (a-CCI) as a tool to predict 30-day post-operative outcome in general surgery patients. ANZ J Surg. 2023;93(1–2):132–8. 36444872 10.1111/ans.18178

[35] Sim MMS, Shiferawe S, Wood JP. Novel strategies in antithrombotic therapy: targeting thrombosis while preserving hemostasis. Front Cardiovasc Med. 2023;10:1272971. 37937289 10.3389/fcvm.2023.1272971 PMC10626538

[36] Tsoupras A, Gkika DA, Siadimas I, Christodoulopoulos I, Efthymiopoulos P, Kyzas GZ. The multifaceted effects of non-steroidal and non-opioid anti-inflammatory and analgesic drugs on platelets: current knowledge, limitations, and future perspectives. Pharmaceuticals (Basel). 2024;17(5):627. 38794197 10.3390/ph17050627 PMC11124379

[37] Hágendorn R, Farkas N, Vincze Á, et al. Chronic kidney disease severely deteriorates the outcome of gastrointestinal bleeding: a meta-analysis. World J Gastroenterol. 2017;23(47):8415–25. 29308001 10.3748/wjg.v23.i47.8415 PMC5743512

[38] Talari K, Goyal M. Retrospective studies - utility and caveats. J R Coll Physicians Edinb. 2020;50(4):398–402. 33469615 10.4997/JRCPE.2020.409

[39] Arai J, Kato J, Toda N, et al. Risk factors of poor prognosis and impairment of activities of daily living in patients with hemorrhagic gastroduodenal ulcers. BMC Gastroenterol. 2021;21(1):16. 33407172 10.1186/s12876-020-01580-w PMC7789673

[40] Xu Y, Siegal DM. Anticoagulant-associated gastrointestinal bleeding: framework for decisions about whether, when and how to resume anticoagulants. J Thromb Haemost. 2021;19(10):2383–93. 34273241 10.1111/jth.15466

[41] Ayonrinde OT, Sanfilippo FM, Schultz C. Prescribing aspirin to older people - where is the line between cardiovascular benefit and upper gastrointestinal bleeding risk? Intern Med J. 2022;52(9):1468–70. 36100571 10.1111/imj.15905

[42] Kamal Ahmed F, Ahmed Mersal F. Abdel Kader mohamed W. Quality of life among elderly people with peptic ulcer at geriatric home. Egypt J Health Care. 2023;14(1):366–79.

[43] Mard SA, Dehkohneh KA, Ahangarpour A. Decreased mRNA expression of cystathionine gamma lyase and H2S concentration in gastric mucosal tissue in diabetic rats. Braz Arch Biol Technol. 2018;61:e18160308.

[44] Liang CM, Hsu CN, Tai WC, et al. Risk factors influencing the outcome of peptic ulcer bleeding in chronic kidney disease after initial endoscopic hemostasis: a nationwide cohort study. Medicine. 2016;95(36):e4795. 27603387 10.1097/MD.0000000000004795 PMC5023910

[45] Thanapirom K, Ridtitid W, Rerknimitr R, et al. Outcome of acute upper gastrointestinal bleeding in patients with coronary artery disease: a matched case-control study. Saudi J Gastroenterol. 2016;22(3):203–7. 27184638 10.4103/1319-3767.182452 PMC4898089

[46] Falconi CA, Junho CVDC, Fogaça-Ruiz F, et al. Uremic toxins: an alarming danger concerning the cardiovascular system. Front Physiol. 2021;12:686249. 34054588 10.3389/fphys.2021.686249 PMC8160254

[47] Kalman RS, Pedrosa MC. Evidence-based review of gastrointestinal bleeding in the chronic kidney disease patient. Semin Dial. 2015;28(1):68–74. 25215610 10.1111/sdi.12301

[48] Islam R, Kundu S, Jha SB, et al. Cirrhosis and coagulopathy: mechanisms of hemostasis changes in liver failure and their management. Cureus. 2022;14(4):e23785. 35518552 10.7759/cureus.23785 PMC9063731

[49] Ardevol A, Ibañez-Sanz G, Profitos J, et al. Survival of patients with cirrhosis and acute peptic ulcer bleeding compared with variceal bleeding using current first-line therapies. Hepatology. 2018;67(4):1458–71. 28714072 10.1002/hep.29370

[50] Infante VM, Román MY, Winograd LR, Ramos CJY, Rodríguez ÁD, Corujo AE. Influence of comorbidity on patients evolution with upper gastrointestinal bleeding from peptic ulcer. Rev Habanera De Cienc Medicas. 2016;15(4):551–62.

[51] Patel P, Nigam N, Sengupta N. Lower gastrointestinal bleeding in patients with coronary artery disease on antithrombotics and subsequent mortality risk. J Gastroenterol Hepatol. 2018;33(6):1185–91. 29156506 10.1111/jgh.14048

[52] Sandek A, Swidsinski A, Schroedl W, et al. Intestinal blood flow in patients with chronic heart failure: a link with bacterial growth, gastrointestinal symptoms, and cachexia. J Am Coll Cardiol. 2014;64(11):1092–102. 25212642 10.1016/j.jacc.2014.06.1179

[53] Henein MY, Vancheri S, Longo G, Vancheri F. The role of inflammation in cardiovascular disease. Int J Mol Sci. 2022;23(21):12906. 36361701 10.3390/ijms232112906 PMC9658900

[54] Pourafkari L, Ghaffari S, Zamani N, et al. Upper gastrointestinal bleeding in the setting of excessive warfarin anticoagulation: risk factors, and clinical outcome. Cor et Vasa. 2017;59(2):e128–33.

[55] Rau W, Hohaus C, Jessen E. A differential approach to form and site of peptic ulcer. Sci Rep. 2019;9:8683. 31213634 10.1038/s41598-019-44893-x PMC6582142

[56] Mille M, Engelhardt T, Stier A. Bleeding duodenal ulcer: strategies in high-risk ulcers. Visc Med. 2021;37(1):52–62. 33718484 10.1159/000513689 PMC7923890

[57] Alsinnari YM, Alqarni MS, Attar M, et al. Risk factors for recurrence of peptic ulcer disease: a retrospective study in tertiary care referral center. Cureus. 2022;14:e22001. 35282517 10.7759/cureus.22001 PMC8907029

[58] Solakoglu T, Koseoglu H, Atalay R, et al. Impact of anti-aggregant, anti-coagulant and non-steroidal anti-inflammatory drugs on hospital outcomes in patients with peptic ulcer bleeding. Saudi J Gastroenterol. 2014;20(2):113–9. 24705149 10.4103/1319-3767.129476 PMC3987151

